# College Commencements

**Published:** 1896-04

**Authors:** 


### COLLEGE COMMENCEMENTS.

The closing exercises of the Southern Medical College, seventeenth session, were held in the Grand Opera House, March 30th. After prayer by Dr. Holderby, the dean of the college, Dr. Jas. B. Baird, made his report. He said that ninety-five students had been in actual attendance during the session, of whom eleven composed the graduating class. The college was the first in this State to adopt the three-years graded course and to exact an educational qualification preliminary to matriculation. These requirements had produced a small class, but had proven a marked benefit in raising the general character of the members. Professor J. McF. Gaston then read a memorial on the late Professor T. S. Powell, paying tribute in well-chosen words to the life and character of this good man and physician. The annual address to the graduates was delivered by Judge Howard Van Epps, President of the Board of Trustees, who chose as the subject of his remarks "The Family Physician." It is impossible in this place to give even an idea of this elegant address. It described in beautiful language the life and hardships of the family physician, and emphasized the wholesome lesson that a physician's first duty was to prepare himself thoroughly for his work.

The valedictorian of the class was Dr. E. L. Awtry, of Atlanta. The first honor was awarded to Dr. R. L. Whipple, of Georgia; the second honor to Dr. E. L. Awtry, of Georgia, and the third honor to Dr. Lucien Lofton, of Atlanta.

---

The commencement exercises of the Atlanta Medical College were held at the Grand Opera House on the evening of March 31, 1896. The stage was adorned with palms, and Wurm's orchestra furnished excellent music. Dr. I. S. Hopkins opened the exercises with prayer.

Dr. W. S. Kendrick, proctor, read his annual report, showing the gratifying attendance, and the good size of the graduating class in spite of this being the first class to graduate under the three-year course. The college has adopted a standard unexcelled by any Southern college, and will continue its progressive course. Rev. J. T. Plunkett delivered the annual address—a well written and well delivered oration.

The valedictory by Dr. R. H. Bell was beautifully written, full of good feeling and affection, and marks Dr. Bell as a master of good composition. Mr. (also Dr.) Frank Park, now a rising attorney, a graduate of the college in medicine, delivered the prizes in a few well chosen words.

Dr. K. L. Reid, of Canada, received first honor. Second honor was shared by Dr. Ned. C. Berry, of Florida, and Dr. J. T. Snider, of Georgia.

The following gentlemen received honorable mention : Drs. W. C. Sistrunk, J. R. Rose, J. S. Grady, S. J. Love, and R. H. Bell.

There were five graduates in the Department of Pharmacy, this being the third year.

The college has safely, and with credit, passed the first commencement under the three-years course, and can look forward to a constantly increasing attendance, a regular upward progression in its standard, and a continuance of its always excellent instruction.

---

THE sixth annual meeting of the Association of Military Surgeons of the United States will convene in Philadelphia on May 12th, 13th and 14th, 1896, and the indications are most flattering for the largest attendance since the organization; many distinguished representatives from the several military services of the United States, as well as a large gubernatorial detail from nearly every State in the Union, have been assured. An interesting program of scientific papers has been arranged to be read and discussed during the day sessions.

ANOTHER popular and prominent Atlanta physician passed away in the person of Dr. Nathan O. Harris, who died from appendicitis March 6th. Dr. Harris was ill a few days with what appeared to be a mild type of the disease, when suddenly symptoms of perforation developed and he was operated upon in a desperate condition. He survived only about thirty-six hours. He was forty-four years of age. No one in Atlanta was more generally known and esteemed. Dr. Harris came to Atlanta when a boy, and had practiced medicine here since 1880. He was ex-president of the Atlanta Society of Medicine, member of our State Association, member of the American Medical Association, and of the National Association of Railway Surgeons. He was local surgeon for the Seaboard Air Line, and surgeon to the Fourth Georgia Battalion. He had been for many years city physician for his ward, and in this connection he did an unusual amount of charity practice, much more than his office demanded. He was a true friend, a good physician, an honest man, and a worthy gentleman.

---

THE work of the Section on Neurology and Medical Jurisprudence at the coming meeting of the American Medical Association at Atlanta promises to be of unusual interest. The desire has been quite generally expressed to have discussions upon the following topics: "The Etiology of Insanity," "Expert Medical Testimony in Disputed Mental Cases," and "Medical Aspects of Crime." It is believed that these topics have a special medical interest at this time, and papers dealing with any phase of these subjects will be very welcome. An urgent invitation is extended to all members, whether they are expecting to be in attendance or not, to present papers and records of cases that have a bearing upon the topics mentioned, or upon any other neurological or medico-legal subject. Any persons who expects to contribute papers or reports to this section will greatly facilitate the business of the section by writing at once to the chairman, T. D. Crothers, M.D., Hartford, Conn., or to the secretary, W. J. Herdman, M.D., Ann Arbor, Mich.

